# Development of prediction models of COVID-19 vaccine uptake among Lebanese and Syrians in a district of Beirut, Lebanon: a population-based study

**DOI:** 10.1136/bmjph-2024-001240

**Published:** 2024-10-09

**Authors:** Marie-Elizabeth Ragi, Hala Ghattas, Hazar Shamas, Jocelyn DeJong, Nada M Melhem, Stephen J McCall, The CAEP Study Group, Aline Germani

**Affiliations:** 1 Center for Research on Population and Health, Faculty of Health Sciences, American University of Beirut, Beirut, Lebanon; 2 Department of Health Promotion, Education, and Behavior, Arnold School of Public Health, University of South Carolina, Columbia, South Carolina, USA; 3 Department of Epidemiology and Population Health, Faculty of Health Sciences, American University of Beirut, Beirut, Lebanon; 4 Medical Laboratory Sciences Program, Division of Health Professions, Faculty of Health Sciences, American University of Beirut, Beirut, Lebanon

**Keywords:** COVID-19, Vaccination, Community Health, Public Health

## Abstract

**Introduction:**

Vaccines are essential to prevent infection and reduce the morbidity of infectious diseases. Previous evidence has shown that migrants and refugees are particularly vulnerable to exclusion and discrimination, and low COVID-19 vaccine intention and uptake were observed among refugees globally. This study aimed to develop and internally validate prediction models of COVID-19 vaccine uptake by nationality.

**Methods:**

This is a nested prognostic population-based cross-sectional analysis. Data were collected between June and October 2022 in Sin-El-Fil, a district of Beirut, Lebanon. The study population included a random sample of Lebanese adults and all Syrian adults residing in areas of low socioeconomic status. Data were collected through a telephone survey. The main outcome was the uptake of at least one dose of the COVID-19 vaccine. Predictors of COVID-19 vaccine uptake were assessed using the Least Absolute Shrinkage and Selection Operator regression for Lebanese and Syrian nationalities in separate models.

**Results:**

Of 2028 participants, 79% were Lebanese, 18% Syrians and 3% of other nationalities. COVID-19 vaccination uptake was higher among Lebanese (85% (95% CI 82% to 86%) compared to Syrians (47% (95% CI 43% to 51%)) (p<0.001); adjusted OR 6.2 (95% CI 4.9 to 7.7). Predictors of uptake of one or more COVID-19 vaccine doses for Lebanese were older age, presence of an older adult in the household, higher education, greater asset-based wealth index, private healthcare coverage, feeling susceptible to COVID-19, belief in the safety and efficacy of vaccines and previous receipt of the influenza vaccine. For Syrians, predictors were older age, male sex, completing school or higher education, receipt of cash assistance, presence of chronic illness, belief in the safety and efficacy of vaccines, previous receipt of the influenza vaccine and possession of a legal residency permit in Lebanon.

**Conclusions:**

These findings indicate barriers to vaccine uptake among Syrian refugees and migrants, including legal residency status. These findings call for urgent action to enable equitable access to vaccines by raising awareness about the importance of vaccination and the targeting of migrant and refugee populations through vaccination campaigns.

**WHAT IS ALREADY KNOWN ON THIS TOPIC**

- Vaccines are essential to prevent infection and reduce the morbidity of infectious diseases, but vulnerable populations may lack access to vaccination campaigns.

**WHAT THIS STUDY ADDS**

- To the best of our knowledge, no studies have compared predictors of COVID-19 vaccine uptake between Syrian refugees and migrants and their Lebanese host communities. This study illustrates a clear difference in vaccine uptake and identified specific predictors of vaccine uptake between nationalities.

**HOW THIS STUDY MIGHT AFFECT RESEARCH, PRACTICE OR POLICY**

- These findings indicate barriers to vaccine uptake among Syrian refugees and migrants, which include possession of legal residency documentation. The findings call for removing barriers and taking public health action to enable access to vaccines for vulnerable groups in future pandemics.

## Introduction

 Vaccines were an essential public health tool to mitigate the impact of the COVID-19 pandemic, but inequalities in vaccine access and uptake exist among subpopulations, particularly refugees. Refugees and migrants are vulnerable to exclusion, stigma and discrimination driven by social attitudes and official legislation,^1^,^3^ which may limit access to vaccination campaigns.^4^,^6^ Studies have shown that refugees and non-native ethnic groups were less willing to receive the COVID-19 vaccine as compared to native populations,^7^,^11^ with racial discrimination and lack of trust in authorities and systems being identified as determinants of COVID-19 vaccine hesitancy.^10 11^ Therefore, the factors that impact vaccine uptake are likely to differ between national and non-national populations.

Lebanon hosts the largest number of refugees per capita in the world, including Syrian refugees who account for approximately one-quarter of the population, many of whom are dispersed in urban residential areas living amidst Lebanese communities.^4 12^ Non-nationals are required to renew their residency permits on an annual basis at significant economic costs, therefore adding to the burden on vulnerable populations.^13^

In Lebanon, the COVID-19 vaccination campaign was initiated in February 2021. It followed the WHO Strategic Advisory Group of Experts on immunisation risk- and age-based approach for prioritisation,^1214^,^16^ and vaccines were available free of charge to all residents of Lebanon by the end of 2021. The COVID-19 national vaccination plan aimed to achieve community immunisation levels of at least 70%–80% by the end of 2022,^12 15 16^ yet there were subpopulations with lower vaccination rates. A survey among older Syrian refugees in Lebanon showed that only 42.5% were vaccinated against COVID-19 by March 2022^17^ while the national average for all residents of Lebanon was 50% by October 2022.^18^ Thus, understanding the demographic variations and predictors of vaccination status between these subpopulations remains important to inform responses to future outbreaks of COVID-19 and future pandemics.

This study aims to determine whether there are differences in COVID-19 vaccine uptake by nationality in a suburb of Beirut, Lebanon and to develop and internally validate prediction models that examine context-specific predictors of COVID-19 vaccine uptake by nationality.

## Methods

### Study design and setting

This is a nested cross-sectional analysis within a multiwave longitudinal study that aims to track vulnerabilities to COVID-19 and other health emergencies in Sin-El-Fil, a suburb of Beirut, Lebanon. The parent study^19^ focused on subpopulations with vulnerabilities that may increase the risk of COVID-19 infection, morbidity or mortality. These included (1) adults aged 60 years or older, (2) pregnant women, (3) adults living in areas of low socioeconomic status and (4) Syrian refugees and migrants.

### Sampling and study population

This was a multistage stratified sampling design using an area-based sampling.^20^ The suburb of Sin-El-Fil was stratified into areas of low and high socioeconomic status, with boundaries defined geographically through stakeholder consultation with the Sin-El-Fil municipality and non-governmental organisations (NGOs). A household listing exercise was carried out in April 2022 whereby all households in Sin-El-Fil were enumerated face to face to complete an eligibility screening survey to determine age, nationality and pregnancy status.^21^ This approach allowed the identification of households and individuals with the specific vulnerability criteria mentioned above. Preconsent was obtained from all households with eligible individuals and phone numbers were collected from consenting households. For the study sample selection, a listing of all eligible household members was generated. A sample of older adults and a sample of adults between 18 and 60 years old were randomly selected using proportionate allocation. Due to small numbers, all Syrian adults and pregnant women were selected. For the present analysis, the study population included all participants living in areas of low socioeconomic status who completed the first wave of data collection conducted between June and October 2022 (n=2045) (see online supplemental figure 1).

Respondents were contacted to complete a computer-assisted telephone survey conducted by a trained data collector and data were entered on SurveyCTO software (Dobility, Cambridge, Massachusetts, USA). Verbal informed consent to participate in an oral telephone interview was obtained from selected respondents and those aged 60 years or older were assessed for capacity to participate using five modified items from the University of California, San Diego, Brief Assessment of Capacity to Consent.^22^

### Data sources

The questionnaire was developed using existing questionnaire modules, contextualised questions and community-identified priorities (see online supplemental table 1).^17^ The survey was created in collaboration with representatives from the municipality of Sin-El-Fil, the Ministry of Public Health and NGOs operating in Sin-El-Fil. The survey tool was drafted in English, translated to Arabic and tested for comprehension prior to deployment. Data were monitored in parallel with data collection for quality assurance. Call-back checks of 5% of the sample were conducted to verify the accuracy of the collected data.

### Outcome measures

COVID-19 vaccine uptake of at least one dose was the primary outcome of interest. Each participant was asked the following questions: ‘Have you received the COVID-19 vaccine?’ and ‘If yes, how many doses did you receive?’. The outcome was presented as binary: ‘received the COVID-19 vaccine’ and ‘did not receive the COVID-19 vaccine’. Additionally, unvaccinated participants were asked if they planned to take the COVID-19 vaccine and the reasons why not.

### Possible predictors

Based on the literature on COVID-19 vaccine uptake, acceptance and hesitancy, several possible predictors for vaccine uptake were identified and included in the model development.^1017 23^,^28^ For Lebanese, these were age (continuous), sex (male/female), education (school not attended/school not completed/school completed/vocational/higher education), household asset-based wealth index (lowest tertile/middle tertile/highest tertile), presence of an older adult in the household (yes/no), presence of chronic illness (yes/no), healthcare coverage (no coverage/private or mutual funds/public funds, NGOs or UN agencies), COVID-19 knowledge (believing COVID-19 is a serious infection: true/false) and risk perception (susceptibility to COVID-19: yes/no), general perceptions of vaccines’ safety and effectiveness (vaccines are safe and/or effective: agree/neither agree or disagree/disagree) and previous receipt of the influenza vaccine (yes/no). The household asset-based wealth index was generated through principal component analysis using household assets that included transportation, communication, home technology and cooking assets. For Syrians, the possible predictors included in the model were age (continuous), sex (male/female), education (school not attended/school not completed/school completed/vocational/higher education), receipt of cash assistance (yes/no), presence of chronic illness (yes/no), healthcare coverage (no coverage/private, mutual or public funds/NGOs or UN agencies), COVID-19 knowledge (believing COVID-19 is a serious infection: true/false) and risk perception (susceptibility to COVID-19: yes/no), general perceptions of vaccines’ safety and effectiveness (vaccines are safe and/or effective: agree/neither agree or disagree/disagree), previous receipt of the influenza vaccine (yes/no), and legal residency status in the country (possession of a legal residency permit for non-nationals) (yes/no). For both Lebanese and Syrian samples, participants were asked if they had been diagnosed with any of the following chronic illnesses: hypertension, type II diabetes, vascular diseases, dyslipidaemia, chronic respiratory diseases, rheumatoid arthritis, chronic kidney diseases or cancer. A variable on the presence of chronic illnesses was subsequently generated (yes/no) where participants had a least one or more chronic illness versus no chronic illness. The missing values were missing at random.^29^ The variable with the highest amount of missing values was at 6.3% and a complete-case analysis was performed.^30^

### Statistical analysis

Frequencies and percentages are presented for categorical variables, and medians with their interquartile range (IQR) for continuous variables. The analysis accounted for the complex design and non-response, and analysis was weighted according to the sampling selection probabilities. The study weight was computed from the product of the probability of selection adjustment in the study sample and the non-response adjustment, that was computed by a response propensity logistic regression model to account for attrition in wave 1 of data collection.^31^ The associations between COVID-19 vaccine uptake and possible predictors were examined by nationality using logistic regression and were presented using unadjusted odds ratios (ORs) and 95% confidence intervals (CIs). All variables were categorical except age, which was found to have a linear association with COVID-19 vaccine uptake.

A Least Absolute Shrinkage and Selection Operator (LASSO) logistic regression model was used to identify the predictors of vaccine uptake for each nationality (Lebanese and Syrian only as the other nationalities sample was limited in numbers),^32 33^ and all corresponding candidate predictors were entered into each model. The optimal model was identified using 10 fold cross-validation. The discrimination of the final models were assesed using the area under the receiver operating curve (AUC), which ranges from 0.5 (representing a discriminative ability equal to chance) to 1.0 (reflecting a perfect discriminative ability between those with and without the outcome). The evaluation of the models’ calibration, which determines the agreement between observed outcomes and predictive probabilities, was performed using calibration plots and slopes. An ideal calibration is illustrated by a diagonal line on the graph with an intercept of 0 and a slope of 1. A slope less than 1 suggests overfitting in the model, meaning that respondents with a high risk of the outcome have overestimated risk predictions, while those with low risk of the outcome have underestimated risk predictions.^34^ Additionally, calibration-in-the-large (CITL) compared the observed number of events and the average predictive risk. A separate logistic regression was conducted to provide ORs and 95% CIs for the selected predictors. A secondary analysis among Syrian refugees and migrants explored the association of legal residency status with vaccination by logistic regression adjusted for confounders, including sex, education and lenght of stay in Lebanon. All analyses were conducted by using Stata/SE statistical software V.18 (STATACorp).

The study followed the Transparent Reporting of a multivariable prediction model for Individual Prognosis or Diagnosis reporting guideline and STrengthening the Reporting of OBservational studies in Epidemiology reporting guideline.^35 36^

## Results

A total of 2028 participants who completed the first wave of data collection and reported their COVID-19 vaccination status were included in this analysis (see online supplemental figure 1), of whom 79% were Lebanese (n=1313), 18% were Syrians (n=658) and 3% were of other nationalities (n=57). The other nationalities included participants from Armenia, Bangladesh, Egypt, Eritrea, Ethiopia, Iran, Iraq, Jordan, Kenya, Kurdistan, Madagascar, Nigeria, Palestine, Philippines, Russia, Senegal, Soudan and Ukraine. Median age of the study sample was 47 years (IQR 32–62, range 18–98) and 45.5% were males.

**Figure 1:**
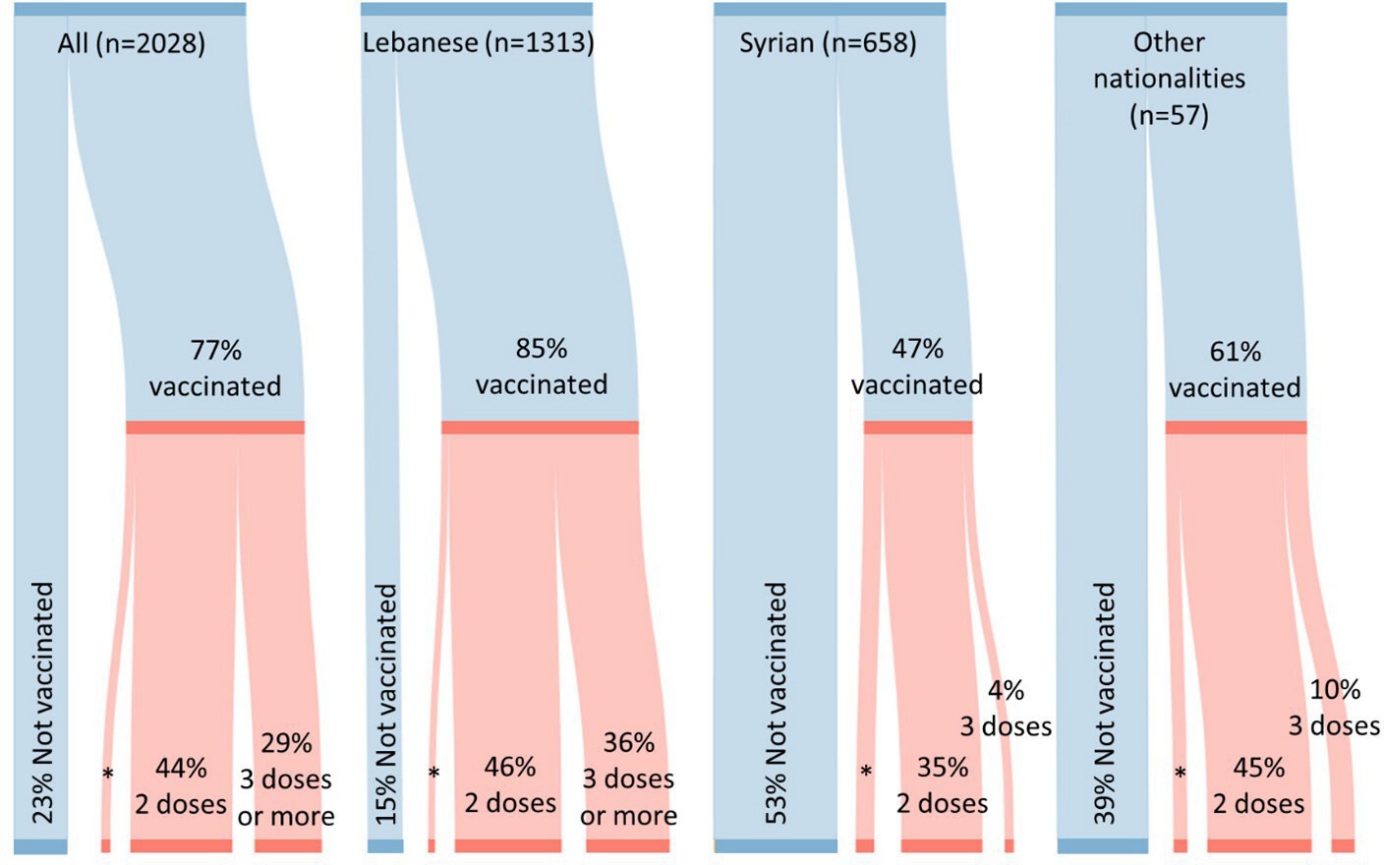
COVID-19 vaccine uptake among study participants. *Uptake of one dose of COVID-19 vaccine was 4% for the total sample, 3% for Lebanese, 8% for Syrians and 6% for other nationalities.

As of October 2022, COVID-19 vaccine uptake among study participants was 77%. There were differences in vaccination status between nationalities (figure 1); less than half (47%) of Syrians had received at least one dose of the COVID-19 vaccine, followed by other nationalities (61%) while a majority of Lebanese (85%) had received at least one dose of the vaccine (p<0.001). In addition, the Lebanese population in this study had received a greater number of vaccine doses with 82% vaccinated with 2 doses or more (vs 39% and 55% for Syrians and other nationalities, respectively). The most common reasons reported for lack of vaccine uptake were: (1) not believing the vaccine is essential and (2) preferring to follow other precautions—by both Lebanese (51% and 25%, respectively) and Syrians (45% and 23%, respectively). For those vaccinated with only one dose (3% of Lebanese and 8% of Syrians), the main reason for not wanting to receive the second dose was fear of side effects for Lebanese (44%), while among Syrians, the highest proportion stated they were still waiting for a second dose (41%) (see online supplemental table 2).

For Lebanese adults, older age, the presence of an older adult in the household, the highest tertile of asset-based wealth index, the presence of at least one chronic illness, having healthcare coverage, belief that COVID-19 is a serious infection, considering themselves susceptible to COVID-19, belief in the safety and efficacy of vaccines in general and previous receipt of the influenza vaccine were all associated with increased odds of receiving the COVID-19 vaccine (table 1).

**Table 1:** Characteristics of the Lebanese population in the study area and associations with COVID-19 vaccine uptake

	Total (n=1313)		Not vaccinated (n=214)		Vaccinated (n=1099)		COVID-19 vaccine uptake
	n	Weighted %	n	Weighted %	n	Weighted %	Weighted Unadjusted OR (95% CI)
Age (years) median (IQR)	1304	52 (35–64)	212	42 (33–57)	1092	54 (36–65)	1.02 (1.01 to 1.03)
Missing	9		2		7		
Sex							
Male	588	(45.0)	93	(43.8)	495	(45.2)	1
Female	725	(55.0)	121	(56.2)	604	(54.8)	0.95 (0.70 to 1.28)
Missing	0		0		0		
Presence of an older adult in the household							
No	565	(40.3)	125	(55.7)	440	(37.5)	1
Yes	743	(59.7)	89	(44.3)	654	(62.5)	2.10 (1.55 to 2.84)
Missing	5		0		5		
Education							
School not attended	103	(8.8)	24	(12.6)	79	(8.1)	1
School not completed	578	(47.6)	89	(43.8)	489	(48.3)	1.72 (1.03 to 2.87)
School completed	174	(14.0)	30	(14.5)	144	(13.9)	1.49 (0.81 to 2.75)
Vocational	117	(9.3)	21	(10.5)	96	(9.1)	1.35 (0.70 to 2.61)
Higher education	251	(20.3)	38	(18.6)	213	(20.6)	1.73 (0.97 to 3.08)
Missing	90		12		78		
Asset-based wealth index							
Lowest tertile	137	(10.5)	31	(14.1)	106	(9.8)	1
Middle	558	(43.4)	100	(48.1)	458	(42.6)	1.27 (0.80 to 2.03)
Highest tertile	587	(46.1)	77	(37.8)	510	(47.6)	1.81 (1.12 to 2.92)
Missing	31		6		25		
Presence of chronic illness							
No	811	(60.5)	153	(70.3)	658	(58.7)	1
Yes	502	(39.5)	61	(29.7)	441	(41.3)	1.66 (1.20 to 2.30)
Missing	0		0		0		
Healthcare coverage							
No coverage	669	(53.8)	145	(69.3)	524	(50.9)	1
Private or mutual funds	78	(6.3)	6	(3.2)	72	(6.9)	2.90 (1.23 to 6.84)
Public funds or NGOs/UN agencies	487	(39.9)	55	(27.5)	432	(42.2)	2.08 (1.48 to 2.93)
Missing	79		8		71		
Believing COVID-19 is a serious infection							
True	1121	(86.6)	168	(79.9)	953	(87.8)	1
False	172	(13.4)	41	(20.1)	131	(12.2)	0.55 (0.37 to 0.82)
Missing	20		5		15		
Consider themselves susceptible to a COVID-19 infection							
No	310	(23.5)	75	(34.9)	235	(21.4)	1
Yes	997	(76.5)	136	(65.1)	861	(78.6)	1.97 (1.42 to 2.71)
Missing	6		3		3		
Vaccines are safe and/or effective							
Agree	1023	(78.3)	86	(39.0)	937	(85.4)	1
Neither agree or disagree	182	(13.7)	56	(26.8)	126	(11.4)	0.19 (0.13 to 0.29)
Disagree	106	(8.0)	71	(34.2)	35	(3.2)	0.04 (0.03 to 0.07)
Missing	2		1		1		
Previous receipt of the influenza vaccine							
No	915	(73.7)	169	(83.4)	746	(71.9)	1
Yes	328	(26.3)	34	(16.6)	294	(28.1)	1.96 (1.31 to 2.93)
Missing	70		11		59		

Data are n and weighted % or median (IQR).

Age was a continuous variable; increasing age was associated with increased odds of receiving the COVID-19 vaccine.

Unadjusted logistic regression analyses were used to examine the association between COVID-19 vaccine uptake and the possible predictors among the Lebanese sample, unadjusted ORs along with their 95% CIs are reported.

NGOs, non-governmental organisations.

For Syrian refugees and migrants, similar to Lebanese, older age, belief that COVID-19 is a serious infection, considering themselves susceptible to COVID-19, belief in the safety and efficacy of vaccines in general, and previous receipt of the influenza vaccine were associated with increased odds of receiving the COVID-19 vaccine; additionally, being male and having a legal residency permit in Lebanon increased the odds of vaccination uptake among Syrians (table 2). In a subgroup analysis among Syrian refugees and migrants, having a legal residency permit in Lebanon, a context-specific factor to Syrian refugees and migrants, doubled the odds of COVID-19 vaccine uptake (adjusted OR 1.7 (95% CI 1.1 to 2.8)).

An unadjusted bivariate analysis for other nationalities found that COVID-19 vaccine uptake was associated with attitudes towards COVID-19 and general vaccination (see online supplemental table 3).

**Table 2:** Characteristics of the Syrian population in the study area and associations with COVID-19 vaccine uptake

	Total (n=658)		Not vaccinated (n=355)		Vaccinated (n=303)		COVID-19 vaccine uptake
	n	Weighted %	n	Weighted %	n	Weighted %	Weighted Unadjusted OR (95% CI)
Age (years) median (IQR)	656	35 (26–42)	353	32 (24–41)	303	37 (28–45)	1.02 (1.01 to 1.04)
Missing	2		2		0		
Sex							
Male	336	(50.3)	154	(41.9)	182	(59.6)	1
Female	322	(49.7)	201	(58.1)	121	(40.4)	0.49 (0.35 to 0.68)
Missing	0		0		0		
Education							
School not attended	93	(14.9)	58	(16.7)	35	(12.9)	1
School not completed	450	(71.2)	248	(72.5)	202	(69.7)	1.25 (0.76 to 2.04)
School completed	51	(8.5)	20	(6.4)	31	(10.8)	2.21 (1.03 to 4.72)
Vocational	17	(2.7)	10	(2.9)	7	(2.5)	1.11 (0.36 to 3.42)
Higher education	17	(2.7)	6	(1.5)	11	(4.1)	3.45 (1.12 to 10.61)
Missing	30		13		17		
Receipt of cash assistance							
No	357	(56.5)	203	(58.9)	154	(53.9)	1
Yes	281	(43.5)	139	(41.1)	142	(46.1)	1.23 (0.88 to 1.71)
Missing	20		13		7		
Presence of chronic illness							
No	533	(79.8)	289	(79.3)	244	(80.4)	1
Yes	125	(20.2)	66	(20.7)	59	(19.6)	0.93 (0.61 to 1.42)
Missing	0		0		0		
Healthcare coverage							
No coverage	500	(77.4)	266	(77.2)	234	(77.6)	1
Private, mutual or public funds	3	(0.4)	2	(0.5)	1	(0.3)	0.56 (0.05 to 6.29)
NGOs/UN agencies	143	(22.2)	79	(22.3)	64	(22.1)	0.98 (0.66 to 1.46)
Missing	12		8		4		
Believing COVID-19 is a serious infection							
True	588	(90.3)	307	(87.7)	281	(93.2)	1
False	60	(9.7)	41	(12.3)	19	(6.8)	0.52 (0.28 to 0.97)
Missing	10		7		3		
Consider themselves susceptible to a COVID-19 infection							
No	161	(23.9)	98	(27.5)	63	(20.0)	1
Yes	495	(76.1)	255	(72.5)	240	(80.0)	1.51 (1.03 to 2.23)
Missing	2		2		0		
Vaccines are safe and/or effective							
Agree	444	(68.4)	183	(51.8)	261	(86.8)	1
Neither agree or disagree	142	(20.8)	106	(29.0)	36	(11.8)	0.24 (0.16 to 0.38)
Disagree	69	(10.8)	64	(19.2)	5	(1.4)	0.04 (0.02 to 0.11)
Missing	3		2		1		
Previous receipt of the influenza vaccine							
No	449	(72.5)	260	(78.4)	189	(65.9)	1
Yes	184	(27.5)	80	(21.6)	104	(34.1)	1.88 (1.30 to 2.71)
Missing	25		15		10		
Possession of a legal residency permit							
No	518	(78.9)	299	(84.4)	219	(72.9)	1
Yes	133	(21.1)	52	(15.6)	81	(27.1)	2.00 (1.31 to 3.05)
Missing	7		4		3		

Data are n and weighted % or median (IQR).

Age was a continuous variable; increasing age was associated with increased odds of receiving the COVID-19 vaccine.

Unadjusted logistic regression analyses were used to examine the association between COVID-19 vaccine uptake and the possible predictors among the Syrian sample, unadjusted ORs along with their 95% CIs are reported.

NGOs, non-governmental organisations.

The final models retained eight predictors of COVID-19 vaccine uptake for both the Lebanese and Syrian populations, respectively. For Lebanese, identified predictors were older age, presence of an older adult in the household, higher education, greater asset-based wealth index, private healthcare coverage, feeling susceptible to COVID-19, belief in the safety and efficacy of vaccines and previous receipt of the influenza vaccine (table 3). Among Syrian refugees and migrants, the predictors were older age, male sex, having completed school or higher education, receipt of cash assistance, presence of chronic illness, belief in the safety and efficacy of vaccines, previous receipt of the influenza vaccine and possession of a legal residency permit in Lebanon (table 4).

**Table 3:** Predictors of vaccine acceptance among Lebanese adults in a district of Beirut

	Codes	Penalised coefficients*	OR (95% CI)†
Age (years)		0.02	1.03 (1.01 to 1.04)
Presence of an older adult in the household			
No	0	–	1
Yes	1	0.46	1.74 (1.16 to 2.62)
Education			
School not attended	1	−0.62	1
School not completed	2	–	1.97 (0.96 to 4.06)
School completed	3	–	2.20 (0.87 to 5.54)
Vocational	4	–	2.23 (0.88 to 5.67)
Higher education	5	0.02	2.47 (1.05 to 5.80)
Asset-based wealth index			
Lowest tertile	1	−0.08	1
Middle	2	–	1.08 (0.62 to 1.89)
Highest tertile	3	0.46	1.78 (0.98 to 3.22)
Healthcare coverage			
No coverage	0	−0.84	1
Private or mutual funds	1	0.19	4.07 (1.51 to 10.94)
Public funds or NGOs/UN agencies	2	–	2.44 (1.59 to 3.74)
Consider themselves susceptible to a COVID-19 infection			
No	0	–	1
Yes	1	0.22	1.34 (0.88 to 2.05)
Vaccines are safe and/or effective			
Agree	1	1.57	1
Neither agree or disagree	2	–	0.20 (0.12 to 0.31)
Disagree	3	−1.50	0.04 (0.02 to 0.07)
Previous receipt of the influenza vaccine			
No	0	–	1
Yes	1	0.04	1.17 (0.72 to 1.91)
Intercept		−0.25	
AUC		0.823 (0.786–0.859)	
C-Slope		1.061 (0.905–1.217)	
CITL		0.004	

A Least Absolute Shrinkage and Selection Operator (LASSO) logistic regression model was conducted. A subsequent logistic regression was run to present ORs and CIs.

* Obtained from the LASSO logistic regression analysis.

† Obtained from logistic regression analysis.

AUC, area under the curve; CITL, calibration-in-the-large; C-Slope, calibration-slope; NGOs, non-governmental organisations.

**Table 4:** Predictors of vaccine acceptance among adult Syrian refugees and migrants in a district of Beirut

	Codes	Penalised coefficients*	OR (95% CI)†
Age (years)		0.02	1.02 (1.01 to 1.04)
Sex			
Male	0	–	1
Female	1	−0.51	0.50 (0.34 to 0.72)
Education			
School not attended	1	−0.03	1
School not completed	2	–	1.12 (0.63 to 1.97)
School completed	3	0.43	2.13 (0.93 to 4.90)
Vocational	4	–	1.13 (0.32 to 4.00)
Higher education	5	0.39	2.36 (0.61 to 9.14)
Receipt of cash assistance			
No	0	–	1
Yes	1	0.19	1.43 (0.98 to 2.90)
Presence of chronic illness			
No	0	–	1
Yes	1	0.02	1.14 (0.70 to 1.87)
Vaccines are safe and/or effective			
Agree	1	1.26	1
Neither agree or disagree	2	–	0.26 (0.16 to 0.41)
Disagree	3	−1.12	0.06 (0.02 to 0.16)
Previous receipt of the influenza vaccine			
No	0	–	1
Yes	1	0.35	1.66 (1.11 to 2.49)
Possession of a legal residency permit			
No	0	–	1
Yes	1	0.51	1.97 (1.18 to 3.29)
Intercept		−1.77	
AUC		0.768 (0.730–0.806)	
C-Slope		1.199 (0.955–1.442)	
CITL		−0.012	

A Least Absolute Shrinkage and Selection Operator (LASSO) logistic regression model was conducted. A subsequent logistic regression was run to present ORs and 95 CIs.

* Obtained from the LASSO logistic regression analysis.

† Obtained from logistic regression analysis.

AUC, area under the curve; CITL, calibration-in-the-large; C-Slope, calibration-slope.

Both models showed good discrimination with an AUC of 0.823 (95% CI 0.786 to 0.859) for the Lebanese sample and 0.768 (95% CI 0.730 to 0.806) for the Syrian sample. The model for the Lebanese population had good calibration with a calibration slope of 1.061 (95% CI 0.905 to 1.217) and CITL of 0.004 (see online supplemental figure 2). The model for the Syrian population had a calibration slope of 1.199 (95% CI 0.955 to 1.442) and CITL of −0.012 (see online supplemental figure 3).

To illustrate the model among Syrian refugees and migrants, an 18-year-old female Syrian with no legal residency permit, who did not attend school, did not receive cash assistance, had no chronic illnesses, did not believe in the safety and efficacy of vaccines and had never taken the influenza vaccine has a 4% chance of having taken the COVID-19 vaccine.^32^

## Discussion

This study revealed inequalities in COVID-19 vaccination status between Syrian and Lebanese adults residing in a low socioeconomic area in Beirut. Overall, 53% of Syrian refugees and migrants had not received any doses of the COVID-19 vaccine compared to 15% among Lebanese. This study also developed and internally validated a prediction model that identified eight predictors of COVID-19 vaccine uptake of at least one dose for the Lebanese and Syrian populations, respectively. This study found that socioeconomic characteristics, demographic factors and health risk perception predicted vaccine uptake among both Syrians and Lebanese. In addition, legal residency status in the country and sex were found to lead to differentials in vaccine uptake within the Syrian population.

The proportion of vaccinated Syrian refugees and migrants in this sample was similar to another study examining COVID-19 vaccination in Syrian refugees aged 50 years or older in Lebanon, which reported 42.5% having received at least one dose of the COVID-19 vaccine by mid-March 2022.^17^ Furthermore, vaccination status of Syrian refugees and migrants in this sample was slightly lower than the 50% national COVID-19 vaccination average recorded at the time of data collection.^18^ In contrast, the proportion of vaccinated Lebanese reported in this study at 85% exceeds this national prevalence, and the majority (82%) were vaccinated with two doses or more, which exceeded the national immunisation target of 70%.^16^ These findings demonstrate large differentials in vaccination uptake by nationality, which indicates barriers to vaccination uptake among Syrian refugees and migrants. Thus, understanding the reasons for not taking the vaccine remains important to design contextualised public health interventions.

A previous study conducted on older Syrian refugees living in Lebanon found similar predictors.^17^ A notable difference was the direction between age and vaccine uptake, which may be due to the larger age range included in the present study.^17^ To the best of our knowledge, there are no prediction models for COVID-19 vaccine uptake among Lebanese adults or younger Syrian adults, as well as scarce literature providing comparative views on COVID-19 vaccination between refugees and the host community in a single setting. In a Jordanian study, Palestinian refugees were found to have lower vaccine uptake and higher rate of facing difficulties during COVID-19 vaccination registration as compared to resident Jordanian citizens.^37^ Furthermore, the same study reported gender, age and education level as being associated with COVID-19 vaccine hesitancy, yet, it did not disaggregate its analysis by nationality.^37^ Accordingly, our study enables the identification of population-specific predictors for refugee and host communities, thus potentially assisting in the development of targeted measures to address the associated barriers.

Some of the identified predictors of COVID-19 vaccine uptake in this study, older age and education level, were common for both Lebanese (national) and Syrian (non-national) populations, which was consistent with studies conducted in the MENA region,^24 38^ the UK,^8^ the USA^9 27 39^ and a global meta-analysis.^26^ In general, older adults have physiological vulnerabilities and comorbidities and thus may perceive themselves at higher risk of infection and severe complications.^24 26 39^ Higher education level was associated with a higher likelihood of vaccine uptake^17^ and a higher willingness to receive the vaccine.^824 26^,^28 38 39^ This may be explained by those with a higher educational level being more aware of the risks of the disease, the importance of vaccination and having the ability to critically appraise vaccine misinformation.^10 39^ It might also relate to a higher level of digital literacy that facilitated access to the online pre-registration platform in Lebanon.^40^ Health and COVID-19 risk beliefs and perceptions, as well as trust in vaccination effectiveness following positive past experiences, also played a role in vaccine acceptance and uptake in line with other studies.^9 24 26 27 39 41^

The socioeconomic indicators identified were in accordance with studies in low-income and middle-income countries that reported low socioeconomic status as a determinant of low willingness to take the COVID-19 vaccine.^28^ Findings also align with other contexts; for example, in the US, low income was associated with lower vaccine uptake and greater vaccine hesitancy.^27 39^ Despite the COVID-19 vaccine being available free of charge to all residents in Lebanon,^16^ this highlights that there may be other barriers to attaining the vaccine specifically for Syrian refugees and migrants.

Legal residency status (having a legal residency permit in Lebanon) was found as a context-specific predictor of COVID-19 vaccine uptake for Syrian refugees and migrants. The majority of the Syrian refugees and migrants (79%) reported not having a legal residency permit; this was likely due to the high costs and requirements relating to obtaining and renewing annual residency permits.^13^ Previous studies in other countries have also shown that the lack of legal residency permits among migrants impacts healthcare access.^42^ Consequently, lack of legal residency may have impacted vaccination uptake as residents of any nationality in Lebanon were required to register on an online government platform,^16^ thus Syrian refugees and migrants, particularly those without documentation, may have worried that registering might lead to arrest or deportation.^6 13 42^

### Strengths and limitations

This study has several strengths and limitations. The study outcome was self-reported which may be prone to information bias; however, the recall of vaccinations among adults was shown to have good agreement with medical record data.^43^ However, the study sample may not be representative of all adults in Lebanon, although it is representative of this particular low socioeconomic suburb in Beirut. The model was slightly underfitted for Syrians and a larger sample will be required for future prediction models among Syrian refugees.

## Conclusion

By October 2022, more than half the Syrian refugees and migrants living in a low socioeconomic area of a suburb of Beirut were still not vaccinated against COVID-19 despite the availability of vaccines free of charge to all residents including non-nationals in Lebanon, as opposed to the majority of Lebanese from the same area having received the vaccine. Predictors for both populations include older age, educational level, socioeconomic status and general attitude towards vaccination. The findings indicate barriers to vaccine uptake among Syrian refugees and migrants, including possession of legal residency documentation. This calls for urgent action to be taken to eliminate barriers and enable equitable access to vaccines for vulnerable populations in future pandemics.

## Supplementary material

10.1136/bmjph-2024-001240 online supplemental file 1

## Data Availability

Data are available on reasonable request.
