# Correction: Development of prediction models of COVID-19 vaccine uptake among Lebanese and Syrians in a district of Beirut, Lebanon: a population- based study

**DOI:** 10.1136/bmjph-2024-001240corr1

**Published:** 2025-07-31

 Ragi M-E, Ghattas H, Shamas H, *et al*. Development of prediction models of COVID-19 vaccine uptake among Lebanese and Syrians in a district of Beirut, Lebanon: a population-based study: *BMJ Public Health* 2024;2:e001240.

This article has been corrected since it was first published. In the funding statement, the grant number for the International Development Research Centre was updated to 1 09 476–001 for proper attribution.

