# Supplementary material for: Development of prediction models of COVID-19 vaccine uptake among Lebanese and Syrians in a district of Beirut, Lebanon: a population-based study
Source: BMJ Public Health. 2024 Oct 9;2(2):e001240. doi: 10.1136/bmjph-2024-001240 (PMC11816316; doi:10.1136/bmjph-2024-001240)
Supplement: online supplemental file 1 [file bmjph-2-2-s001.pdf]

Supplementary material

Supplementary Table 1 – Survey questions

Variable	Question	Options
**Sex**	Specify Respondent's gender	1-Male<br>2-Female
**Month of Birth**	Specify Respondent's year of birth	YYYY
**Year of Birth**	Specify Respondent's month of birth	MM
**Nationality**	Confirm Respondent's nationality	1-Lebanese<br>2-Syrian<br>3-Other. If other, specify:
**Older adults in the household**	a. What is the total number of people who live in the HH?<br>b. Of those, how many are 60+ years old?	____
**Education**	Have you ever attended school?	1-Yes<br>0-No<br>88-Don't know/ Refuse to answer
	If yes, what is your level of education?	1-Elementary -> specify last grade completed<br>2-Preparatory -> specify last grade completed<br>3-Secondary -> specify last grade completed<br>4-Vocational<br>5-University -> specify whether graduated or not<br>6-Post graduate (masters, PhD) -> specify whether graduated or not<br>88-Don't know/ Refuse to answer
**Legal residency in Lebanon**	For Syrian nationalities:<br>Do you have regularized residency in Lebanon?	1-Yes<br>0-No<br>88-Don't know/ Refuse to answer
**Chronic illnesses**	Have you ever been told by a health care professional that you have any of the following chronic illnesses? (select all that apply)	1-Hypertension<br>2-Diabetes<br>3-Vascular Diseases (heart diseases or stroke)<br>4-Chronic Respiratory Diseases

		5-Rheumatoid Arthritis<br>6-Chronic kidney diseases<br>7-Cancer<br>8-Dyslipidemia (high blood cholesterol or high blood triglycerides)<br>0-None<br>88-Don't Know/ Refuse to answer
**Health Care Coverage**	What are the sources of healthcare coverage funding that you have? (select multiple)	1-Private insurance<br>2-Insurance with orders/syndicates<br>3-Mutual Funds<br>4-National Social Security Fund (NSSF)<br>5-Ministry of Public Health (MOPH)<br>6-Army<br>7-Other public funds:<br>Civil Servants Cooperative (CSC)<br>Internal Security Forces (ISF)<br>General Security Forces (GSF)<br>State Security Forces (SSF)<br>8-NGOs/ UN agencies<br>9-Nothing<br>77-Other. If other, specify:<br>88-Don't know/ Refuse to answer
**COVID-19 knowledge**	COVID-19 is a serious infection	1-True<br>2-False<br>88-Don't know
**COVID-19 Perceived risk**	Do you consider yourself susceptible to a COVID-19 infection?	1-Yes<br>0-No<br>99-Refuse to answer
**Vaccines**	I think vaccines are safe	1-Agree<br>2-Neither agree or disagree<br>3-Disagree<br>99-Refuse to answer
	I think vaccines are effective	1-Agree<br>2-Neither agree or disagree<br>3-Disagree

		99-Refuse to answer
	Have you ever received the flu vaccine?	1-Yes<br>0-No<br>99-Refuse to answer
**COVID-19 vaccination**	Have you received the COVID-19 vaccine?	1-Yes<br>0-No<br>88-Don't know<br>99-Refuse to answer
	If vaccinated, how many doses did you receive?	1-One<br>2-Two<br>3-Three<br>4-Four<br>5-More than four. If more than 4, please specify:<br>99-Refuse to answer
	If one dose, why?	1-Medical condition/Doctor advise<br>2-Don't want to, fear of side-effects<br>3-Think one dose is enough<br>4-Waiting for second dose<br>77-Other. If other, specify :<br>99-Refuse to answer
	If not vaccinated, do you plan to receive the COVID-19 vaccine?	1-Yes<br>0-No<br>88-Don't know<br>99-Refuse to answer
	If you do not plan to receive the COVID-19 vaccine, why not?	1-Too new I would rather wait until I/we know more<br>2-I would rather continue to follow precautions than take a vaccine<br>3-I do not believe the vaccine is essential<br>4-I do not believe COVID-19 requires a vaccine<br>77-Other. If other, specify:<br>88-Don't know<br>99-Refuse to answer
**Assets**	Does anyone in your household (including yourself) have any of the following assets that are FUNCTIONAL?	

	Car	1-Yes<br>0-No<br>88-Don't know<br>99-Refuse to answer
	Motorbike/Scooter	
	Van / Pick-up Truck	
	Bicycle	
	Small gas stove for cooking	
	Oven	
	Refrigerator	
	Microwave	
	Iron	
	Air conditioning	
	Heater/heating stove (electric, diesel, wood etc.)	
	Water heater	
	Washing machine	
	TV	
	Computer	
	Mobile phone	
	Access to the internet (3G/Wifi)	
**Assistance**	Have you received any cash assistance in the last 6 months?	1-Yes<br>0-No<br>88-Don't know<br>99-Refuse to answer

Supplementary Table 2 – Reasons for COVID-19 vaccine uptake hesitancy in our study sample across nationalities

	Total		Lebanese		Syrian		Other Nationalities		P-value
	n	*Weighted %*	*n*	*Weighted %*	*n*	*Weighted %*	*n*	*Weighted %*	
**For those unvaccinated, the main reason why they did not receive the COVID-19 vaccine**									
Plan to register on platform	56	(8.5)	10	(4.5)	43	(13.2)	3	(15.7)	
Prefer other precautionary measures	121	(23.0)	50	(24.9)	71	(23.5)	0	(0.0)	
Do not believe vaccine is essential	252	(49.3)	103	(51.5)	137	(44.7)	12	(59.3)	0.013
Do not believe COVID-19 requires a vaccine	46	(9.2)	17	(8.3)	25	(9.0)	4	(20.5)	
Other*	52	(10.0)	21	(10.8)	30	(9.6)	1	(4.5)	
**Reasons only one dose of the COVID-19 vaccine was taken**									
Medical condition/Doctor advise	9	(7.6)	3	(6.4)	6	(10.7)	0	(0.0)	
Not wanting to for fear of side effects	31	(35.3)	17	(43.9)	14	(24.8)	0	(0.0)	
Think one dose is enough	25	(27.5)	13	(29.0)	10	(19.7)	2	(67.1)	0.129
Waiting for second dose	27	(28.3)	8	(20.7)	18	(41.1)	1	(32.9)	
Other	2	(1.3)	0	(0.0)	2	(3.7)	0	(0.0)	

A P-value less than 0·05 was considered statistically significant.
**Other reasons include: Mistrust, fear or hesitancy because of controversial information and the vaccine being new ; Medical reasons*

Supplementary Table 3 - Characteristics of other nationalities in our study area and associations with COVID-19 vaccine uptake

	Total (n=57)		Not vaccinated (n=22)		Vaccinated (n=35)		COVID-19 vaccine uptake
	n	Weighted %	n	Weighted %	n	Weighted %	Weighted Unadjusted OR [95% CI]
**Age (years) Median**(IQR)	57	35 (30-46)	22	31 (27-35)	35	40 (34-52)	1.06 [0.97-1.16]
**Sex**							
Male	18	(33.0)	9	(42.9)	9	(26.5)	1
Female	39	(67.0)	13	(57.1)	26	(73.5)	2.08 [0.63-6.89]
Missing	0		0		0		
**Presence of an older adult in the household**							
No	47	(80.3)	18	(79.3)	29	(81.0)	1
Yes	10	(19.7)	4	(20.7)	6	(19.0)	0.90 [0.21-3.85]
Missing	0		0		0		
**Education**							
School not attended	18	(33.5)	6	(29.6)	12	(36.2)	1
School not completed	21	(43.5)	11	(54.4)	10	(35.8)	0.54 [0.14-2.12]
School completed	3	(6.3)	1	(5.2)	2	(7.2)	1.14 [0.08-16.88]
Vocational	1	(2.4)	0	(0.0)	1	(4.1)	.
Higher Education	7	(14.3)	2	(10.8)	5	(16.7)	1.27 [0.17-9.43]
Missing	7		2		5		
**Assets-based wealth index**							
Poorest tertile	37	(66.2)	13	(62.6)	24	(68.5)	1
Middle	11	(19.3)	5	(22.8)	6	(17.1)	0.69 [0.16-2.93]
Richest tertle	8	(14.5)	3	(14.6)	5	(14.4)	0.90 [0.17-4.70]
Missing	1		1		0		
**Receiving cash assistance**							
No	49	(87.7)	20	(95.0)	29	(83.1)	1
Yes	7	(12.3)	1	(5.0)	6	(16.9)	3.86 [0.40-37.56]
Missing	1		1		0		
**Presence of chronic illness**							
No	47	(81.6)	19	(84.9)	28	(79.5)	1
Yes	10	(18.4)	3	(15.1)	7	(20.5)	1.45 [0.31-6.75]
Missing	0		0		0		
**Believing COVID-19 is a serious infection**							
True	45	(82.7)	15	(71.9)	30	(89.3)	1

False	10	(17.3)	6	(28.1)	4	(10.7)	0.31 [0.07-1.38]
Missing	2		1		1		
**Consider themselves susceptible to a COVID-19 infection**							
No	22	(39.9)	12	(57.7)	10	(28.4)	1
Yes	35	(60.1)	10	(42.3)	25	(71.6)	3.43 [1.06-11.07]
Missing	0		0		0		
**Vaccines are safe and/or effective**							
Agree	37	(68.2)	7	(35.8)	30	(86.2)	1
Neither agree or disagree	8	(13.9)	5	(25.3)	3	(7.6)	0.12 [0.02-0.74]
Disagree	9	(17.9)	7	(38.9)	2	(6.2)	0.07 [0.01-0.42]
Missing	3		3		0		
**Previous receipt of the flu vaccine**							
No	39	(79.3)	18	(97.7)	21	(68.0)	1
Yes	12	(20.7)	1	(2.3)	11	(32.0)	20.02 [2.16-185.73]
Missing	6		3		3		

Data are n and weighted % or median (IQR), OR=odds ratio. CI=confidence interval.
Age was a continuous variable.
Unadjusted logistic regression analyses were used to examine the association between COVID-19 vaccine uptake and the possible predictors among other nationalities, unadjusted odds ratios (ORs) along with their 95% confidence intervals (CIs) are reported.
The wealth index was generated from the presence in the household of functional transportation, communication, home technology and cooking assets.
The chronic illnesses assessed included hypertension, type II diabetes, vascular diseases, dyslipidemia, chronic respiratory diseases, rheumatoid arthritis, chronic kidney diseases, cancer.

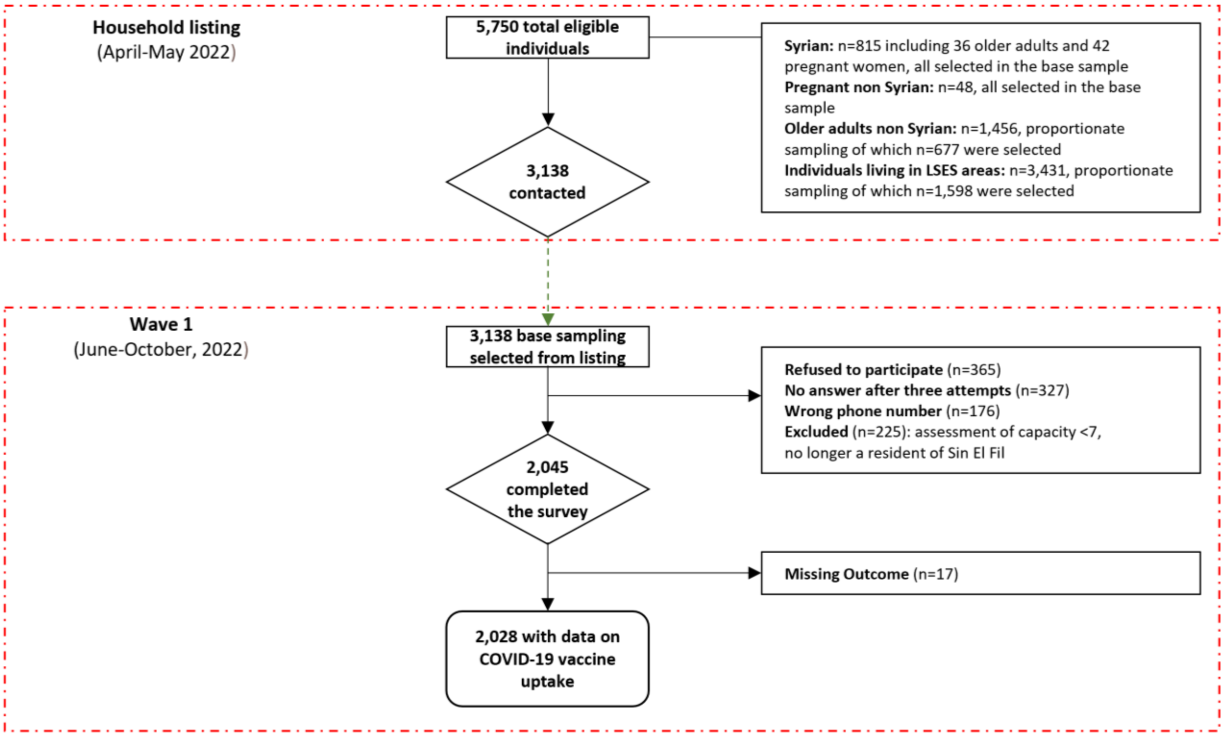

Supplementary Figure 1. Flow diagram of the study population who completed the survey at wave 1

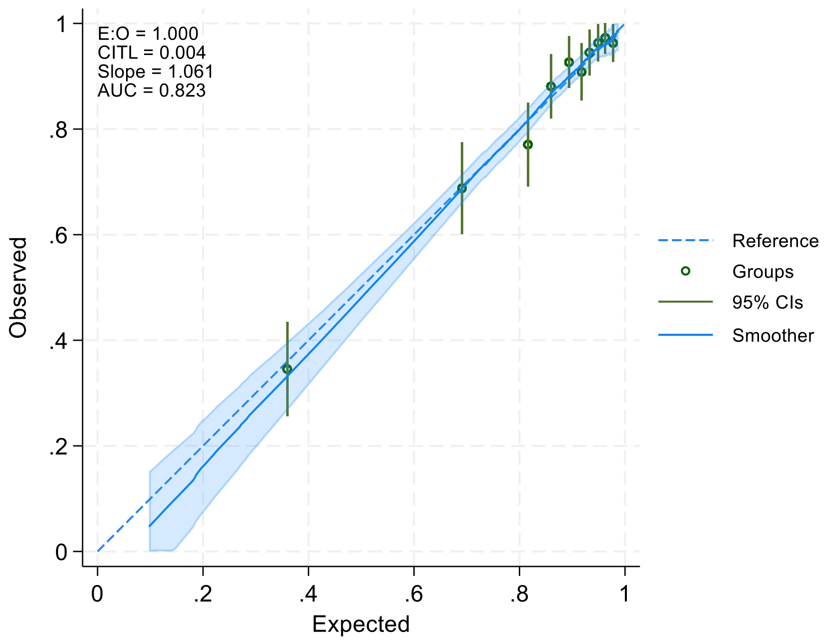

Supplementary Figure 2. Final model performance for the Lebanese sample
E:O= Expected to Observed ratio (calibration) ; CITL= calibration-in-the-large ; Slope=Calibration-slope ; AUC= Area Under the Curve (discrimination)

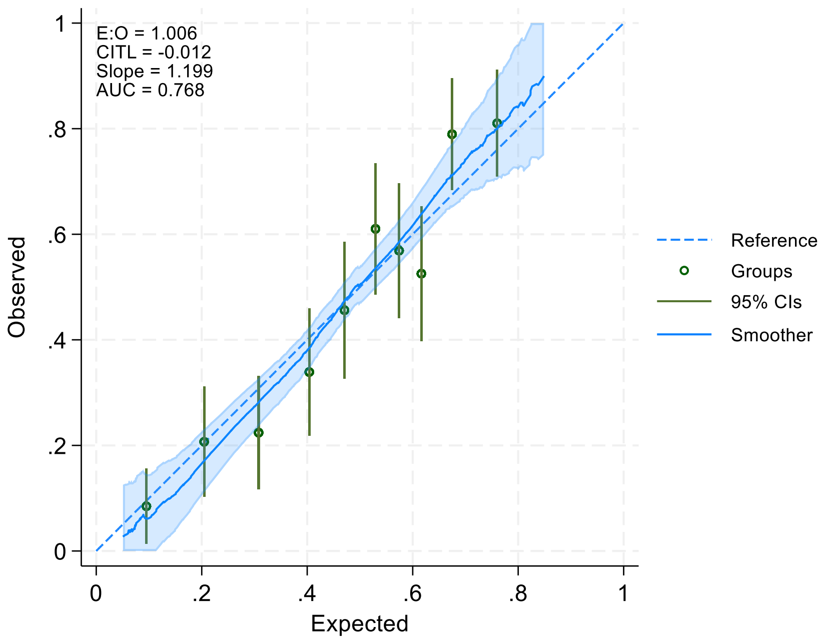

Supplementary Figure 3. Final model performance for the Syrian sample
E:O= Expected to Observed ratio (calibration) ; CITL= calibration-in-the-large ; Slope=Calibration-slope ; AUC= Area Under the Curve (discrimination)
